# Tissue- and development-specific glycosylation states of the natriuretic peptide receptors guanylyl cyclase-A (GC-A) and GC-B

**DOI:** 10.1186/1471-2210-11-S1-P52

**Published:** 2011-08-01

**Authors:** Dieter Müller, Mirjam Hildebrand, Jörn Lübberstedt, Ralf Middendorff

**Affiliations:** 1 Institute of Anatomy and Cell Biology, University of Giessen, Germany

## Background

The natriuretic peptides (NPs) atrial NP (ANP), B-type NP (BNP) and C-type NP (CNP) elicit cellular effects by binding to transmembrane proteins with guanylyl cyclase (GC) activity, referred to as GC-A (representing the common receptor for ANP and BNP) and GC-B (the CNP receptor). GC-A and GC-B are structurally closely related proteins with very similar cDNA-deduced molecular masses in the range of 115 kDa, but their native molecular weights are significantly (by up to 20 kDa) higher due to glycosylation.

Differential glycosylation of cell surface receptors can affect folding, trafficking, ligand binding and/or agonist-induced signal transduction. Moreover, specific temporal expression patterns of carbohydrate structures are frequently involved in developmental processes. Hitherto, however, the glycosylation states of GC-A and GC-B *in vivo* are largely unknown, since currently available data are based to a great extent on studies with receptor-transfected cell lines.

This investigation aimed to characterize the sizes and posttranslational modifications of native GC-A and GC-B in different tissues of adult rats and mice. Based on increasing evidence for specific activities of NPs and their receptors in the developing brain (1-3), we examined the glycosylation of GC-A and GC-B during postnatal brain development.

## Methods and results

Western blot (as well as receptor/ligand crosslinking) experiments and treatments with carbohydrate-digesting enzymes served to investigate the sizes and posttranslational modifications of the two receptors. Due to marked tissue-specific differences in N-linked glycosylation, mean apparent molecular masses of both rat and mouse GC-A range from 119 kDa in brain to values between 124 (lung) and 132 (heart) kDa in peripheral tissues. The reduced glycosylation in brain seems to be central nervous system (CNS)-specific, since the pituitary, which contains both CNS and CNS-unrelated tissue, co-expresses the CNS-typical (119 kDa, reference: olfactory bulb) and the peripheral (up to 130 Da) forms of GC-A.

GC-B sizes differed in the same manner between peripheral organs, indicating equal tissue-dependent influences on oligosaccharide processing at GC-A and GC-B. A higher (by 3 kDa) total amount of N-linked carbohydrates suggests usage of an additional consensus N-glycosylation site (7 instead of 6 in the case of GC-A). The existence of region-specific GC-B size variants in cerebellum and olfactory bulb discriminates this receptor from GC-A in the adult brain.

Both GC-A and GC-B (but not two other glycosylated membrane proteins examined) are hyperglycosylated at N-linked sites during early postnatal brain development. At postnatal day 1, the vast majority of GC-B (but not GC-A) contain additionally an O-linked carbohydrate modification.

Affinity labelling and membrane GC assays did not reveal marked glycoform-associated effects on ligand binding or agonist-induced cGMP production.

## Conclusion

The data uncover tissue-, development- and receptor-specific glycosylation states. Functional consequences remain to be elucidated.
